# Draft Genome Sequences of Three Sub-Antarctic *Rhodococcus* spp., Including Two Novel Psychrophilic Genomospecies

**DOI:** 10.1128/genomeA.00898-17

**Published:** 2017-09-07

**Authors:** Akhikun Nahar, Anthony L. Baker, Michael A. Charleston, John P. Bowman, Margaret L. Britz

**Affiliations:** a Tasmanian Institute of Agriculture, University of Tasmania, Hobart, Australia; b School of Physical Sciences, University of Tasmania, Hobart, Australia; c Faculty of Veterinary and Agricultural Sciences, University of Melbourne, Melbourne, Australia

## Abstract

The draft genome sequences of three sub-Antarctic *Rhodococcus* sp. strains—1159, 1163, and 1168—are reported here. The estimated genome sizes were 7.09 Mb with a 62.3% GC content for strain 1159, 4.45 Mb with a 62.3% GC content for strain 1163, and 5.06 Mb with a 62.10% GC content for strain 1168.

## GENOME ANNOUNCEMENT

*Rhodococcus*, a genus in the order *Actinomycetales*, is ubiquitous in terrestrial ecosystems (1). Several species are biotechnologically important for their capability to biodegrade pollutants (2–4) and for their oleaginous nature (5–7). Here, we report the whole-genome sequences of *Rhodococcus* sp. strains 1159, 1163, and 1168.

To identify potential mycolic acid-producing bacteria, the University of Tasmania Antarctic culture collection was screened for lysozyme-resistant bacteria, which revealed these three strains originally isolated in 2001 from soil and detritus on Macquarie Island (54°36′S, 158°54′E). Strain 1159 is off-white to pale yellow when cultured on nutrient agar or minimal salt broth, and its optimal growth temperature is 30°C. The other two strains are yellow-orange when cultured similarly, with fastest growth occurring at 20°C.

High-molecular-weight genomic DNA was extracted following a modified extraction method originally described by Lévy-Frébault et al. (8) and whole-genome shotgun (WGS) sequenced using Illumina MiSeq technology by Macrogen (South Korea). Totals of 5,846,628, 6,354,712, and 6,661,398 reads with 1,752,144,208, 1,898,840,255, and 1,989,884,802 bases were obtained for strains 1159, 1163, and 1168, respectively. Raw sequences were *de novo* assembled using the AbySS sequence assembler (9), and 112, 43, and 97 contigs with lengths of ≥200 bp were obtained for strains 1159, 1163, and 1168, respectively. Genome annotation was performed using RAST (10) and the NCBI Prokaryotic Genome Annotation Pipeline (11).

The estimated genome size of strain 1159 was 7.09 Mb with a 62.3% GC content and 6,546 coding sequences (CDSs), 6,385 coding genes, and 161 pseudogenes. These features are similar to those of *R. erythropolis* strain JCM 6824 (WGS, GenBank accession number BBLL00000000), which has a genome size of 7.02 Mb with a 62.3% GC content, encoding 6,608 genes and 6,372 proteins. However, the estimated genome sizes of strains 1163 and 1168 were 4.45 and 5.06 Mb with 62.3% and 62.10% GC contents, respectively. There were 4,042 CDSs, 3,956 coding genes, and 86 pseudogenes predicted for strain 1163 and 4,617 CDSs, 4,463 coding genes, and 154 pseudogenes predicted for strain 1168. These features most closely resembled those of *R. fasciens* F7 (NZ_LFDS00000000), which had a 5.24-Mb genome with a 64.7% GC content and 4,819 proteins.

Genetic similarities were calculated against the phylogenetically close species using the average nucleotide identity (ANI) tool of IMG/M (12). Strain 1159 was 98.7%, 95.6%, and 95.42% genetically similar to *R. erythropolis* 339MFSha3.1 (Ga0101996), *R. qingshengii* BKS 20-40 (Ga0032278), and *R. baikonurensis* JCM 18801 (Ga0128325), respectively. As such, this strain was identified as *R. erythropolis*. Strains 1163 and 1168, however, form a separate phylogenetic clade in the 16S rRNA gene neighbor-joining tree, demonstrating, respectively, 78.43% and 78.62% similarities with *R. yunnanensis* (NZ_BCXH00000000) and 78.06% and 77.95% similarities with *R. fascians* 02-815 (Ga0125507). Strains 1163 and 1168 were calculated to be 93.7% similar to each other, below the ANI speciation point of 95%. These two strains are therefore suggested to represent novel species.

The RAST SEED Viewer identified a large number of genes involved in carbohydrate and lipid metabolism. Genes for aromatic compound metabolism were identified, indicating biotechnological potential.

### Accession number(s).

These whole-genome shotgun projects have been submitted to DDBJ/EMBL/GenBank under the accession numbers MJVD00000000, MKKX00000000, and MKKY00000000.
